# Supplementary material for: Identification, classification, and expression profile analysis of heat shock transcription factor gene family in Salvia miltiorrhiza
Source: PeerJ. 2022 Dec 5;10:e14464. doi: 10.7717/peerj.14464 (PMC9745953; doi:10.7717/peerj.14464)

**Table S4. Sequence and distribution information of all motifs in *SmHSFs***

| **Gene Name** | **subgroup** | **DBD** | **HR-A** | **HR-B** | **NLS** | **AHA** |
| --- | --- | --- | --- | --- | --- | --- |
| SmHSF1 | A6 | 62-160 | 166-206 | 208-236 |  |  |
| SmHSF2 | A7 | 35-133 | 143-183 | 188-216 | 217-239 |  |
| SmHSF3 | A2 | 40-138 | 148-188 | 193-221 |  |  |
| SmHSF4 | B2 | 23-121 | 160-200 |  |  |  |
| SmHSF5 | A6 | 57-155 | 161-201 | 206-234 |  |  |
| SmHSF6 | B4 | 22-120 | 171-211 |  |  |  |
| SmHSF7 | A7 | 41-139 | 147-187 | 192-220 | 221-243 |  |
| SmHSF8 | B3 | 19-117 | 142-182 |  |  |  |
| SmHSF9 | A2 | 29-127 | 148-188 | 193-221 |  |  |
| SmHSF10 | B2 | 24-122 | 167-207 |  |  |  |
| SmHSF11 | A1 | 15-113 | 129-169 | 174-202 |  | 444-458 |
| SmHSF12 | A6 | 39-137 | 147-187 | 192-220 | 221-243 |  |
| SmHSF13 | A6 | 39-100 | 101-141 | 146-174 | 175-197 |  |
| SmHSF14 | A5 | 17-115 | 121-161 | 166-194 |  | 429-443 |
| SmHSF15 | A3 | 47-145 | 147-187 | 192-220 |  |  |
| SmHSF16 | A4 | 11-109 | 118-158 | 163-191 |  | 362-379 |
| SmHSF17 | A9 | 25-124 | 151-191 |  |  |  |
| SmHSF18 | A9 | 23-122 | 130-170 |  |  |  |
| SmHSF19 | A9 | 14-113 | 140-180 |  |  |  |
| SmHSF20 | B4 | 17-114 | 127-167 |  |  |  |
| SmHSF21 | B4 | 22-120 | 156-196 |  |  |  |
| SmHSF22 | A4 | 11-109 | 118-158 | 163-191 |  | 358-372 |
| SmHSF23 | A1 | 30-128 | 145-185 | 190-218 |  |  |
| SmHSF24 | B4 | 32-94 | 145-185 |  |  |  |
| SmHSF25 | A8 | 11-109 | 122-162 | 167-195 |  |  |
| SmHSF26 | B2 | 23-121 | 124-164 |  |  |  |
| SmHSF27 | A6 | 20-118 | 133-173 | 178-206 | 207-229 |  |
| SmHSF28 | A4 | 12-110 | 121-161 | 166-194 |  | 335-349 |
| SmHSF29 | B4 | 19-122 | 137-177 |  |  |  |
| SmHSF30 | C | 22-120 | 135-175 |  |  |  |
| SmHSF31 | C | 16-114 | 121-161 |  |  |  |
| SmHSF32 | A3 | 94-192 | 201-241 | 246-274 |  |  |
| SmHSF33 | A6 | 64-162 | 168-208 | 213-241 |  |  |
| SmHSF34 | B3 | 18-116 | 139-179 |  |  |  |
| SmHSF35 | B1 | 7-105 | 150-190 |  |  |  |

Motif 1


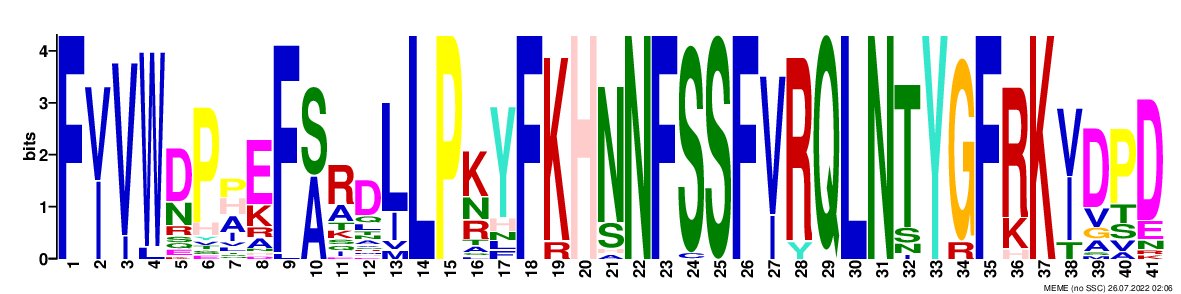


Motif 2


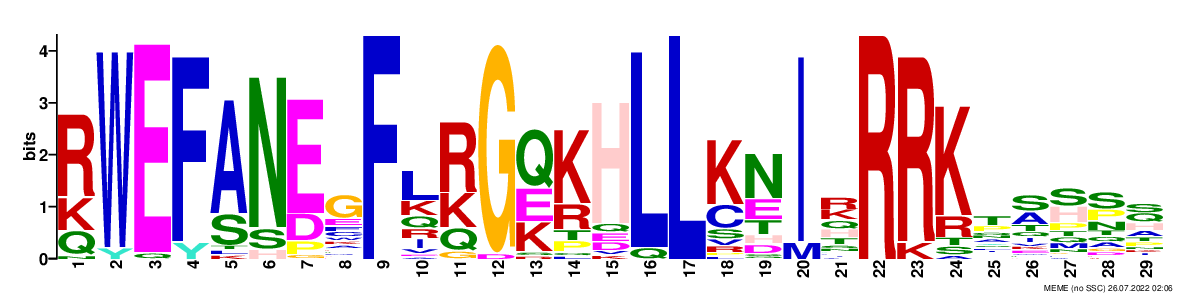


Motif 3


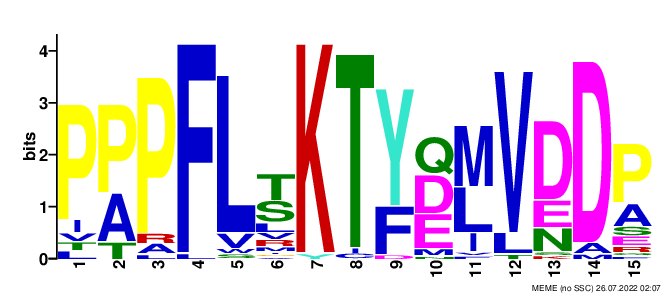


Motif 4


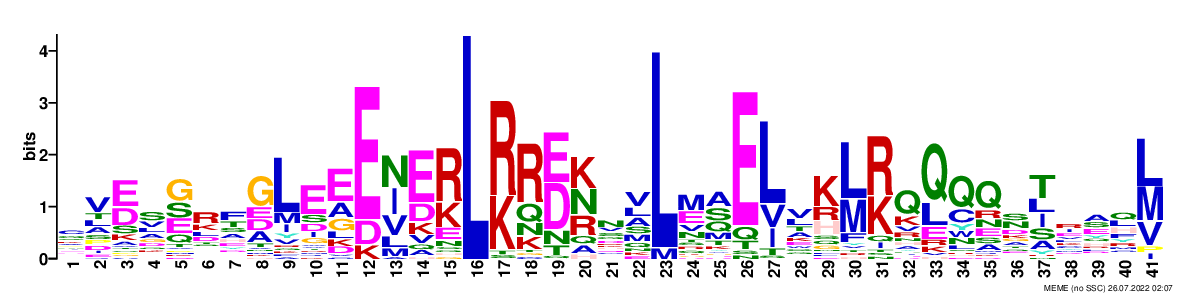


Motif 5


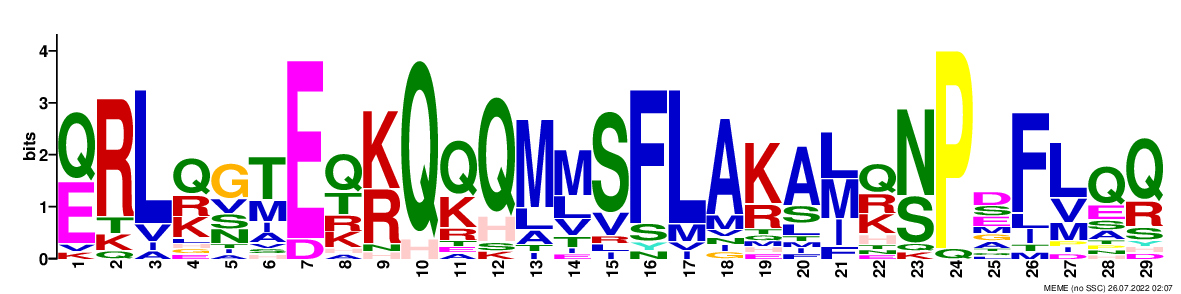


Motif 6


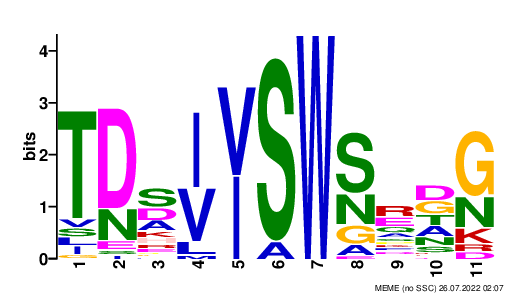


Motif 7


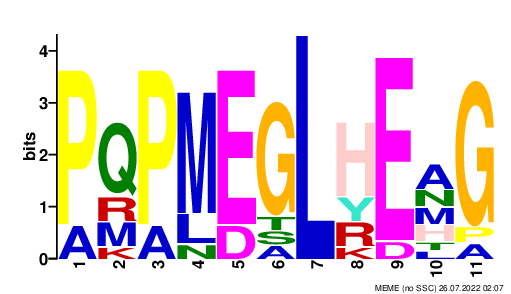


Motif 8


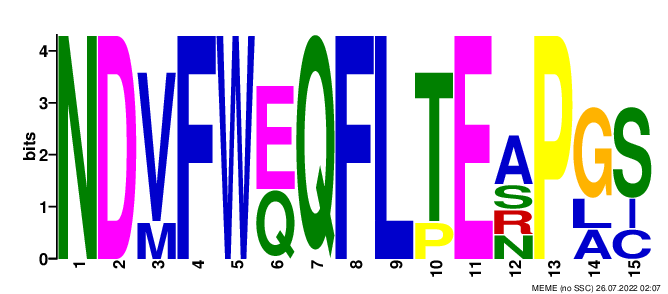


Motif 9


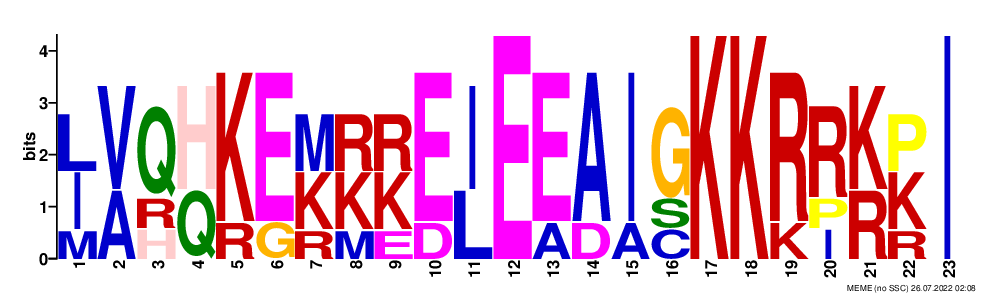


Motif 10


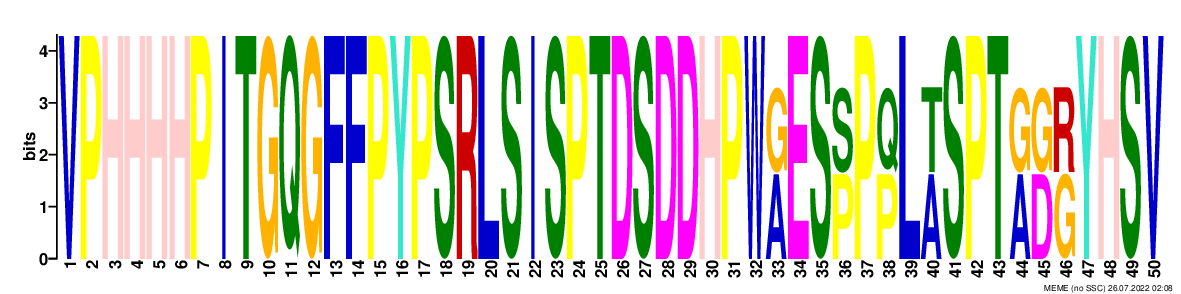

Supplement: Supplemental Information 4 — Including the motif distribution of the SmHSF genes and 10 motif sequences analyzed by MEME [file peerj-10-14464-s004.docx]
